# Dr. Arthur John Sharp

**Published:** 1910-06-18

**Authors:** 


We have to record the death of Dr. Arthur John Sharp, F.R.C.S., at Nottingham, in his forty-third year. He was an exhibitioner of Lincoln College, Oxford, took his M.B. (with first-class honours) and his B.S. in 1892, his diploma in 1893, and graduated M.D. of London in 1894. Two years later he became a F.R.C.S. At one time he was resident obstetric assistant and house physician at Guy's Hospital, and clinical assistant at the Evelina Hospital. He contributed also to the medical papers.

